# Morphological and genetic characterization of novel *Sarocladium spinificis* strains in association with *Coccidioides posadasii*

**DOI:** 10.1128/spectrum.00689-25

**Published:** 2025-12-29

**Authors:** Marcus de M. Teixeira, Sarah A. Ahmed, Heather L. Mead, Sybren de Hoog, Nathan Wiederhold, Jason E. Stajich, Daniel R. Matute, George R. Thompson, Bridget M. Barker

**Affiliations:** 1 Núcleo de Medicina Tropical, Faculdade de Medicina, Universidade de Brasília, Brasília, Brazil; 2 The Pathogen and Microbiome Institute, Northern Arizona University 3356, Flagstaff, Arizona, USA; 3 Department of Microbiology, College of Medicine, Kuwait University 37603, Kuwait City, Kuwait; 4 Translational Genomics Research Institute 525768 , Flagstaff, Arizona, USA; 5 Radboudumc-CWZ Centre of Expertise for Mycology 6034, Nijmegen, the Netherlands; 6 University of Texas Health Science Center at San Antonio 14742, San Antonio, Texas, USA; 7 Department of Microbiology & Plant Pathology, Institute for Integrative Genome Biology, University of California 8790, Riverside, California, USA; 8 Department of Biology, University of North Carolina at Chapel Hill 2331, Chapel Hill, North Carolina, USA; 9 Department of Medicine/Division of Infectious Diseases and Medical Microbiology and Immunology, University of California Davis 8789, Davis, California, USA; Brown University, Providence, Rhode Island, USA

**Keywords:** *Sarocladium spinificis*, fungal pathogens, comparative genomics, opportunistic infections, *Coccidioides posadasii*, antifungal resistance, microbial interactions

## Abstract

*Sarocladium* is a fungal genus containing diverse members with roles as pathogens of plants and animals, endophytes, and biocontrol agents. This study characterizes two *Sarocladium spinificis* strains isolated from patients diagnosed with coccidioidomycosis. Morphological, phylogenetic, and genomic analyses confirmed their classification within the *Sarocladium* genus, closely related to *S. spinificis*. Both strains exhibited mycelial growth at 24°C and 37°C, with distinct morphological features. Comparative genomic analysis identified unique orthologous clusters and functional gene families associated with diverse carbon utilization and potential pathogenicity. Notably, these strains’ genomes contain many secreted proteins, carbohydrate-activated enzymes, and peptidases, highlighting their metabolic versatility. Co-cultivation experiments revealed that *S. spinificis* strains CA16 and CA18 both inhibited the growth of *Coccidioides posadasii*, suggesting antagonistic interactions and potential antifungal properties. These findings underscore the ecological and clinical significance of *S. spinificis* and its impact on microbial communities, advancing our understanding of its diversity, pathogenic potential, and antifungal capabilities.

**IMPORTANCE:**

Accurate identification and characterization of emerging fungal pathogens are essential for improving diagnosis and treatment. *Sarocladium spinificis*, though not widely recognized as a human pathogen, exhibits traits suggesting a potential role in opportunistic infections. This study provides the first detailed morphological, phylogenetic, and genomic characterization of clinical *S. spinificis* isolates, highlighting its ability to survive at body temperature and its antifungal resistance. Additionally, we demonstrate that these strains inhibit *Coccidioides posadasii*, the agent of Valley fever, suggesting ecological competition or antifungal properties. These findings contribute to understanding fungal interactions in clinical and environmental settings, with implications for fungal pathogenesis and antifungal strategies. By uncovering new aspects of *S. spinificis* biology, this work expands knowledge of *Sarocladium* species in human health and lays the foundation for future research on its ecological role, pathogenic potential, and therapeutic applications.

## INTRODUCTION

The taxonomic recognition of *Sarocladium* as a new fungal genus was initiated by Gams and Hawksworth in 1975, who identified this organism as the cause of sheath-rot disease in rice (1). The genus *Sarocladium* was formally recognized in 2015, initially composed of 10 species, and has gained attention for its diverse ecological roles and emerging clinical relevance (2). This genus was initially characterized as *Acremonium*, but after a careful morphological examination, *Sarocladium* and *Acremonium* can be distinguished based on distinctive microscopic characteristics and multi-locus sequencing type methods (3, 4). *Sarocladium* exhibits elongated phialides emerging individually on vegetative hyphae or on conidiophores with sparse or repeated branching, abundant adelophialides, and elongated conidia. In contrast, *Acremonium* typically features mainly unbranched or poorly basitonously branched conidiophores, variable-shaped conidia (subglobose, obovate, and ellipsoidal), and the usual absence of adelophialides (2). Based on the phylogenetic species concept, multi-locus sequencing typing (MLST) revealed that *Sarocladium*, *Parasarocladium*, *Chlamydocillium*, and *Polyphialocladium* form a unique family named Sarocladiaceae within the Hypocreales (4, 5). *S. oryzae* and *S. attenuatum* were designated as type species of *Sarocladium* sharing the ability to cause sheath rot in rice plants (2). The genus *Sarocladium* encompasses a diverse array of ecological functions with at least 37 species within its classification (6). These species play diverse roles, from saprotrophs decomposing organic matter to pathogens infecting plants and humans. They also form endophytic associations, engage in mycoparasitism, and influence soil ecosystem dynamics (3, 7, 8). In addition, *Sarocladium* has gained recognition as a biocontrol agent against various plant diseases due to its ability to produce secondary metabolites that suppress fungal growth (9–11).

Notably, *Sarocladium* infections in humans are on the rise. At least 309 cases of human infections caused by *Acremonium* and *Sarocladium* have been described (8). *Sarocladium* species, including *Sarocladium kiliense*, *S. hominis*, *S. bactrocephalum*, *S. bifurcatum*, *S. pseudostrictum*, *S. subulatum*, and *S. terricola*, have been associated with opportunistic infections in humans (2, 8). These species can grow at 37°C, a characteristic shared by many human fungal pathogens (2, 4). Meningitis associated with *Sarocladium* has been described in patients with immunological disorders (12–15). An outbreak of bloodstream infections attributed to *S. kiliense* emerged following the administration of contaminated antinausea medication in Colombia and Chile between 2013 and 2014. Through comprehensive whole-genome analysis, investigators identified a shared source of infection, confirming the presence of the fungus in contaminated vials of the medication (16). Lastly, recent reports have identified *Sarocladium* infections in individuals diagnosed with COVID-19 (17, 18). Contrastingly, *Sarocladium* are also abundant commensal fungi in the human mycobiome. Patients diagnosed with ulcerative colitis had lower levels of *Saccharomyces* and *Sarocladium* taxa and increased burden of *Candida* compared to healthy controls (19). Understanding the implications of opportunistic or concurrent infections, versus an association with a healthy microbiome, is crucial for ensuring correct diagnosis and treatment strategies.

While investigating the fungal etiology underlying several cases of coccidioidomycosis, our study unexpectedly identified two isolates of *Sarocladium spinificis*, revealing a previously undocumented occurrence of this species in clinical specimens. Delving deeper, we undertook comprehensive phylogenetic and genomic analysis as well as co-cultivation experiments with *Coccidioides*. Comparative genomic analysis reveals unique genomic traits in *Sarocladium* species. Intriguingly, our findings demonstrated the suppressive effect of *Sarocladium spinificis* on *Coccidioides posadasii*, which could be a path for antifungal development.

## MATERIALS AND METHODS

### Fungal isolation and phenotypic characterization

*Sarocladium* sp. isolates CA16 (CBS 144516) and CA18 (CBS 144517) were recovered at a clinical laboratory in Madera, CA, USA. Strain CA16 was obtained from a skin (lip) lesion, while CA18 was isolated from a left leg wound, both from patients in Madera. These isolates were from patients with Accuprobe-confirmed cases of coccidioidomycosis. Samples were sent to the Fungal Testing Lab (FTL) in San Antonio, TX, USA, for antifungal testing in 2012 and 2013. These samples were then sent to Northern Arizona University (NAU) in 2015. Monosporic isolation procedures were conducted within a biosafety level 3 facility at NAU due to the presumed etiological agent *Coccidioides* classified as risk group 3. Mycelia were cultivated on 2× GYE (2% glucose, Thermo Fisher Scientific, USA; 1% yeast extract, BD Difco) agar at 25°C for a duration of 10 days. Macroscopic morphological assessment involved inoculating the fungus on various media, including 2% malt extract agar (MEA, BD Difco), potato dextrose agar (PDA, BD Difco), oatmeal agar (OA, BD Difco), and 2× GYE agar plates, which were then incubated for 7 days at 25°C. Additionally, 2× GYE plates were incubated at 37°C for 7 days, and morphology was evaluated (Fig. S1). For microscopic characterization, the slide culture method was employed, wherein mycelia from macrocolonies were inoculated with a sterile loop into small agar blocks of 2× GYE, MEA, PDA, and OA. These blocks were covered with sterile glass coverslips (2 × 2 cm) and incubated for 1 week in a moist chamber. After growth, the coverslips were mounted in lactic acid and visualized with Zeiss AXIO Scope A1 (Carl Zeiss, Germany) equipped with differential interface contrast objects. Micrographs were taken with Axiocam 208 camera and processed in ZEN Microscopy Software (Carl Zeiss), and contrast and sharpness were adjusted in Adobe Photoshop (Adobe Inc., USA).

The competition assay between *C. posadasii* Δcts2/Δard1/Δcts3 (NR-166), which can be handled in a biosafety level 2 and *S. spinificis* CA16 and CA18 strains, was performed on a 2× GYE petri dish at 25°C since both species grow well on those conditions. We harvested conidia of both *C. posadasii* and *S. spinificis* using the protocols described previously (20). Next, we inoculated 10^7^ *C. posadasii* NR-166 conidia cells based on hemocytometer counts verified with viability testing and uniformly spread onto 2× GYE plates. We let the plates dry, and 10^6^ conidia cells of *S. spinificis* CA16 strain or CA18 were plated in the center of the petri dish. We incubated the plates in the dark at 25°C for 10 days and captured images using an Olympus SZ-11 camera.

The susceptibility of the two *S*. *spinificis* isolates to antifungal drugs was conducted following the CLSI M38Ed3 reference guidelines (21). In brief, RPMI-1640 (0.165 M MOPS, pH 7.0, without bicarbonate) served as the growth medium. Stock solutions at 100× concentrations of amphotericin B, fluconazole, itraconazole, and voriconazole were prepared in DMSO with further dilutions made in RPMI such that the final DMSO concentration within the *in vitro* assay was 1% vol/vol. Minimum inhibitory concentrations (MICs) of amphotericin B, posaconazole, itraconazole, and voriconazole were read at 100% inhibition of growth compared to drug-free control after 48 hours of incubation at 35°C, and fluconazole MICs were measured at 50% growth inhibition. Samples were tested in 2012/2013 and 2025.

### DNA extraction and molecular characterization

We extracted high molecular weight DNA from approximately 500 mg of freshly harvested mycelial tissue of the two *S*. *spinificis* strains, obtained 7 days post-inoculation on 2× GYE agar plates. For DNA isolation, we employed the UltraClean Microbial DNA Isolation Kit (MoBio, Qiagen). We achieved fungal cell lysis through vigorous agitation in MicroBead tubes from the kit using a vortex adapter tube holder (MoBio, Qiagen) for 15 min. To assess DNA quality and quantity, we performed electrophoresis on a 0.8% agarose gel stained with SYBR Safe (Thermo Fisher Scientific) and measured absorbance using a NanoDrop ND-1000 system (Thermo Fisher Scientific). Given the initial diagnosis of the isolates as *Coccidioides* spp., we used a real-time PCR-based research lab test targeting a specific repetitive region unique to *Coccidioides* genome, which yielded negative results (22). We used universal primers targeting the internal transcribed spacer (ITS) region to identify the fungus, specifically ITS1 and ITS4 primers (23) at NAU and FTL. Approximately 20 ng of DNA served as input for the PCR amplifications of the ITS region as follows: 25 µL of MyFi Mix (Bioline; Meridian Bioscience, USA), 0.2 µM of each primer in final concentration, DNA template, and ultrapure water to a final volume of 50 µL. The thermocycling steps were initial denaturation for 2 min at 95°C, followed by 35 cycles of 15 s at 95°C, 15 s at 55°C, and 30 s at 72°C, and a final elongation step of 5 min at 72°C. PCR fragments were purified using the QIAquick PCR Purification kit (Qiagen, USA) and sequenced in an ABI 3130xl Genetic Analyzer instrument (Thermo Fisher Scientific) using the BigDye (v.3.1) Terminator Cycle Sequencing kits (Thermo Fisher Scientific). Electropherograms were examined using the Phred algorithm with a tool hosted on our local server. Sequences were compared against the National Center for Biotechnology Information (NCBI) nucleotide database using the Basic Local Alignment Search Tool (BLAST) algorithm (24).

### Genome sequencing, assembly, and annotation

Genome sequencing was performed using 1 μg of DNA per isolate for paired-end sequencing. DNA was fragmented via sonication, followed by size selection at 500 bp. Library preparation followed the Kapa Biosystems Kit protocol (Kapa Biosystems, Woburn, MA, USA), incorporating an 8 bp index for multiplexing. Library quality was assessed using quantitative PCR (qPCR) on a 7900HT System (Life Technologies Corporation, Carlsbad, CA, USA) with a Kapa Library Quantification Kit (Kapa Biosystems). Sequencing was carried out in an Illumina MiSeq instrument under high-output (v.3) 600-cycle mode, generating 2 × 300 bp paired-end reads. Demultiplexing was performed using standard tools in BaseSpace.

The genomes of *S. spinificis* CA16 and CA18 were assembled and annotated (PRJNA1092660). We used the AAFTF/0.4.1 pipeline (25) for assembly, and the fungal genome annotation pipeline, funannotate (v.1.8) (Palmer and Stajich 2020) for annotation. We also identified and masked repetitive DNA elements using TANTAN (26). For gene prediction, we used the funannotate “predict” command, incorporating evidence-based and *ab initio* (structure-based) prediction pathways. To train the *ab initio* predictors Augustus (27), GlimmerHMM (28), and SNAP (29), we retrieved conserved gene models from the sordariomycetes_odb10 BUSCO database (30). Additionally, we also used GeneMark-ES with the option tailored for fungal genomes to further enhance ORF prediction and improve the accuracy of gene model annotation (31). We inferred a weighted consensus gene structure via EVidenceModeler (32), with a weight of 2 assigned to Augustus HiQ models and a weight of 1 for others. We also predicted tRNAs using tRNAscan-SE (33). We filtered out genes with a length less than 50 amino acids and those identified as transposable elements for all downstream analyses.

We investigated gene family expansions by comparing the six available *Sarocladium* genomes against multiple functional annotation databases. Protein function was assigned using eggNOG 5.0 (34), Pfam (v.36.0) (35), and InterProScan (v.96.0) (36). Additionally, specific functional categories, including carbohydrate-degrading enzymes (CAZy/dbCAN v.11.0 [37]), proteases (MEROPS v.12.0 [38]), and secreted proteins (SignalP v.5.0 [39]), were analyzed to gain further insights into metabolic capabilities and potential pathogenicity factors. We completed Gene Ontology (GO) analyses as part of the annotation funannotate predict step (40). Finally, we predicted biosynthetic gene clusters in the two *Sarocladium* genomes using fungiSMASH, a fungal-genome-specific version of antiSMASH (v.6.0) (41).

### Phylogenetic trees

Next, we explored the phylogenetic relationships of the two sequenced isolates with other *Sarocladium* isolates. We used two approaches: MLST and phylogenomic analysis. We described the protocols to generate input data and ran the phylogenetic analyses as follows.

1. MLST-based tree: Most fungal data available for phylogenetics exist in the form of sequences of a few loci. In particular, the 28S large subunit ribosomal RNA gene (28S), internal transcribed spacer region (ITS1–5.8S–ITS2), translation elongation factor 1-alpha, second largest subunit of RNA polymerase II, and actin are often used in fungal phylogenetics (4, 42, 43). We retrieved these five genetic markers from the CA16 and CA18 genomes using nucleotide BLAST analysis and aligned them to another 54 related taxa (Table S1) using the MAFFT online server (44). It is worth noting that some isolates of *S. spinificis* were previously classified as distinct species before phylogenetic analyses clarified their taxonomic placement (4). Manual curation was performed, and phylogenetically informative sites from each genetic marker were collected using the ClipKIT (v.1.3.0) function smart-gap (45). Phylogenetic trees were inferred using the maximum likelihood method implemented in IQ-TREE2 (46). The nucleotide substitution model was automatically selected using the -m TEST function, which determines the best-fitting model based on the Akaike Information Criterion (AICX [47]). To assess branch support, we performed ultrafast bootstrap approximation (UFBoot [48]) and approximate likelihood ratio tests (aLRT [49]). The resulting tree was visualized using FigTree software (v.1.4) (http://tree.bio.ed.ac.uk/software/figtree/). We used *Parasarocladium wereldwijsianum* strain NL19095011 as an outgroup to root the tree.
2. Phylogenomic tree: To generate phylogenetic trees, we used the PHYling pipeline (https://github.com/stajichlab/PHYling), which extracts phylogenetically conserved markers and constructs species trees from annotated genomes. The marker-based approach was complemented by a genome-wide phylogenetic analysis with a smaller taxonomic representation, providing an additional perspective on evolutionary relationships among the studied taxa. We used the set of Benchmarking Universal Single-Copy Orthologs (BUSCO) listed in the fungi_odb10 database (30). We retrieved the protein models from five previously published *Sarocladium*: *S. implicatum* TR *-* GCA_021176775.1, *S. kiliense* ZJ-1 - GCA_030734395.1, *S. strictum* F4-1 - GCA_030435815.1, *S. oryzae* JCM 12450 - GCA_001972265.1, and *S. brachiariae* HND5- GCA_008271525.1. We used the hmmsearch tool from HMMER (v.3.3.2) (50) to retrieve BUSCO orthologous genomic loci. We generated protein alignments using the hmmbuild function of HMMER (v.3.3.2) (50) and the ClipKIT (v.1.3.0) tool (45) using the smartgap function to eliminate spurious positions. *Tolypocladium ophioglossoides* strain CBS100239 was used as the outgroup (51). We applied genome-wide phylogenetic analysis using the genealogical concordance approach in the IQ-TREE 2 software (46). We used the -m TEST function to infer the best nucleotide and protein alignment models. To assess branch support, we calculated 1,000 ultrafast bootstraps (48), the aLRT (49), and approximate Bayes test for each node (52). The resulting phylogenetic trees were visualized using FigTree (v.1.4.4) software (https://tree.bio.ed.ac.uk/software/figtree/).

### Comparative genomics

We used the genomic data from CA16 and CA18 and the previously sequenced *Sarocladium* genomes to identify gene family expansions. The funannotate (v.1.8) “compare” function was applied to the six *Sarocladium* species to assess trends among the annotated protein categories. Heat maps were produced from those uneven counts of protein families or classes with standard deviation greater than 1.0. We visualized shared and unique orthologous clusters in *Sarocladium* using Orthoveen3 (53).

## RESULTS

### Clinical cases

Two *Sarocladium spinificis* isolates, CA16 and CA18, were initially identified as *Coccidioides* spp. at a clinical laboratory in Madera, CA. The samples were sent to the FTL at the University of Texas Health San Antonio, TX, USA, because patients were not responding to antifungal therapy. Strain CA16 was obtained from a patient presenting with a persistent ulcerative lesion on the lower lip, characterized by chronic inflammation. The lesion had an indurated border and displayed progressive erosion, raising suspicion of a fungal etiology. Similarly, isolate CA18 was recovered from a chronic, non-healing wound on the left lower leg of a different patient, also from Madera, CA. The wound initially developed following minor trauma but failed to heal despite standard wound care and antibiotic therapy. Biopsy and mycological analysis led to the identification of a filamentous fungus, presumed *Coccidioides*, as the etiological agent using the Accuprobe assay on the total DNA from the isolate sample. The samples were sent to NAU for a *Coccidioides*-focused genomic study. However, after storage in glycerol for 2 years, the qPCR-based assays using a *Coccidioides*-specific clinical research lab test yielded negative results (22).

### Morphological and molecular characterization of CA16 and CA18 strains

**Fig 1:**
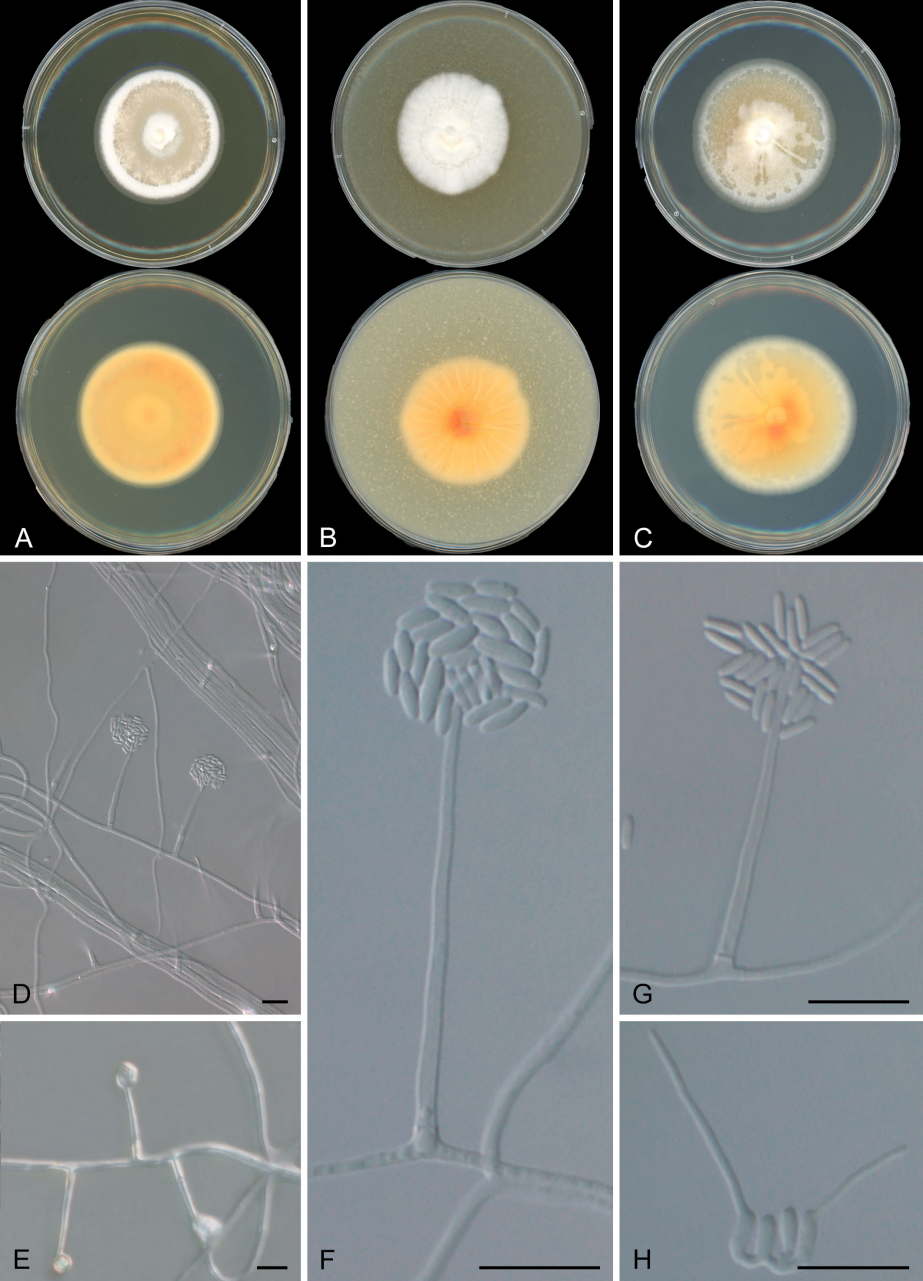
Growth and micromorphology of *Sarocladium spinificis* strains CA16 and CA18. Both strains exhibited growth at 24°C on MEA (**A**), OA (**B**), and PDA (**C**), displaying similar micromorphology with minor variations in colony morphology. Microscopically, hyphae are septate, branching, smooth, and hyaline**,** with some forming a rope-like appearance. Conidiophores are phialidic and undifferentiated**,** with erect, mostly solitary phialides that are rarely branched, cylindrical, and possess inconspicuous collarettes. Conidia are produced in slimy-head formations, appearing cylindrical to ellipsoidal and measuring 2–7 µm in length (**D–H**). Scale bars = 10 µm

**Fig 2:**
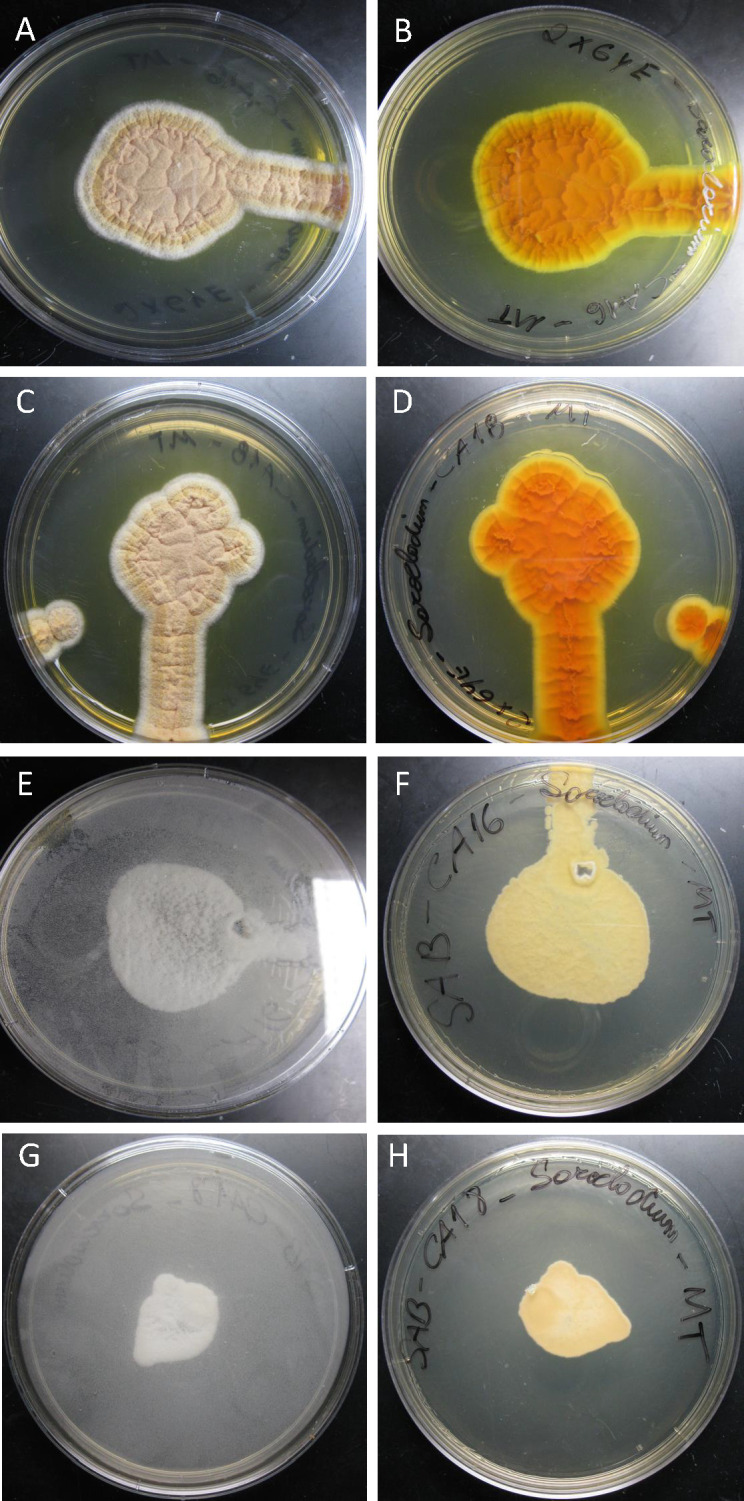
Growth characteristics of *Sarocladium spinificis* strains CA16 and CA18 at 24°C and 37°C. (**A and B**) Macrocolonies of CA16 and (**C and D**) CA18 grown on 2X-GYE agar at 24°C for 15 days, measuring 44 and 36 mm, respectively. Colonies were irregular and wrinkled, with a pale orange center, white margins, and an orange reverse, while both strains produced a soluble yellow pigment diffused in the agar. (**E and F**) CA16 and (**G and H**) CA18 grown at 37°C for 15 days, reaching 42 and 24 mm, respectively. Colonies at this temperature were irregular, cottony white, with a pale to yellowish reverse, and showed reduced growth with no pigmentation.

Both CA16 and CA18 strains grew at 24°C and 37°C on PDA, MEA, and OA showing similar morphology with little phenotypic variability (Fig. 1A through C; Fig. S1). At both temperatures, the strains grew in filamentous form. Colonies were circular, regular, flat, whitish, or creamy buff, sometimes radially or slightly sulcate. In all media, yellow to orange pigment was produced at room temperature on the reverse side of the petri dish. However, at 37°C, the colonies produced no pigmentation and grew slower (Fig. 2). The colonies of CA16 (Fig. 2A and B) and CA18 (Fig. 2C and D) grown on 2× GYE media at 24°C for 15 days measured 44 and 36 mm, respectively. Colonies were irregular and wrinkled, with a pale orange center, white margins, and an orange reverse, while both strains produced a soluble yellow pigment diffused in the agar (Fig. 1A through C; Fig. S1). At 37°C, CA16 (Fig. 2E and F) and CA18 (Fig. 2G and H) reached 42 and 24 mm after 15 days of growth, respectively, forming irregular, cottony white colonies with a pale to yellowish reverse (Fig. S1). Microscopically, hyphae were septate, branching, smooth, and hyaline, some arranged in rope-like appearance; conidiophores were phialidic and undifferentiated; phialides were erect, mostly solitary, rarely branching, cylindrical, with inconspicuous collarettes, producing conidia in slimy-heads; conidia were cylindrical or ellipsoidal, measuring 2–7 µm (Fig. 1D through H). Both isolates showed moderate resistance to antifungals initially, but current testing indicates higher MICs. Table 1 shows the MICs for what was most likely the two *Coccidioides* isolates initially received and tested by the clinical laboratory in 2012 and 2013, and the two S. *spinificis* isolates recovered after storage in glycerol (2025).

**TABLE 1:** MICs of four antifungal agents for *Sarocladium spinificis* isolates CA16 and CA18 for initial isolates and after glycerol storage^*a*^*^,^*^*b*^

Antifungal agent	CA16 2012	CA18 2013	CA16 2025	CA18 2025
Amphotericin B	0.125	0.06	8	8
Fluconazole	16	16	>64	>64
Itraconazole	0.5	1.0	>16	>16
Voriconazole	0.25	0.06	1	1

^*a*^ All measurements are in μg/mL (mg/L). The MIC values from 2012 and 2013 most likely represent those against the *Coccidioides* isolates identified by DNA probe in 2012 and 2013, while those from 2025 were tested against *S. spinificis*.

^*b*^ MIC, minimum inhibitory concentration.

**Fig 3:**
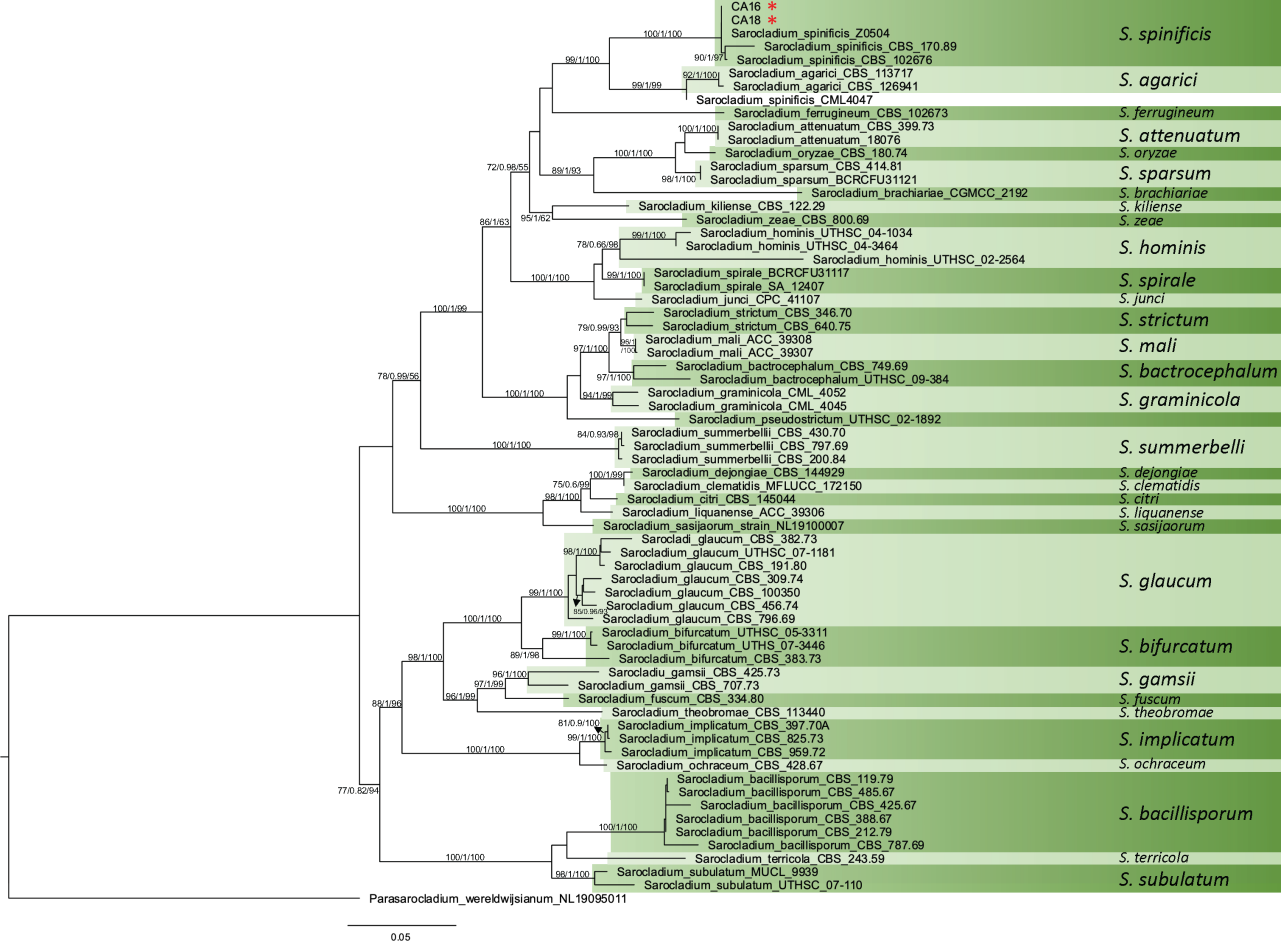
MLST-based phylogenetic tree of *Sarocladium spinificis*. Maximum likelihood (ML) phylogenetic tree inferred from concatenated sequences of five loci (LSU, ITS, translation elongation factor 1-alpha, second largest subunit of RNA polymerase II, and actin) retrieved from *S. spinificis* strains CA16 and CA18, along with 54 related taxa (Table S1). Phylogenetic analyses were performed using IQ-TREE2, with model selection determined by the Akaike information criterion. Branch support was assessed using ultrafast bootstrap approximation and approximate likelihood ratio tests. The tree was visualized in FigTree v.1.4, with *Parasarocladium wereldwijsianum* strain NL19095011 used as the outgroup for rooting. Branch lengths in the ML tree are proportional to the number of nucleotide substitutions per site, reflecting genetic divergence between taxa.

Next, we amplified and sequenced the universal fungi barcode rDNA locus ITS for both CA16 (MF784843.1) and CA18 (MF784844.1) strains from glycerol stocks, confirmed at both NAU and FTL. Nucleotide BLAST analyses revealed that those two isolates were 99.82% identical to *S. spinificis* strain Y. H. Yeh I0317 (MK336486.1), isolated from a Beach Morning Glory (*Ipomoea pes-caprae*) plant in Taiwan. The maximum likelihood MLST-based phylogenetic tree indicated that both CA16 and CA18 clustered with three isolates of *S. spinificis*. The branch leading to these five isolates had high levels of support (bootstrap and aLRT, Fig. 3). Two *S*. *spinificis* strains, CBS 170.89 and CBS 102676, form a discrete dyad, but we have no power to resolve the relationships between the other three strains of the group (CA16, CA18, and Z0504). This clade is sister to a clade formed by *S. agarici* and a sixth isolate of *S. spinificis* (CML4047). The sister relationship of *S. spinificis* and *S. agarici* is concordant with previous results (4), but the position of CML4047 isolate is puzzling, as it groups with *S. agarici*.

**Fig 4:**
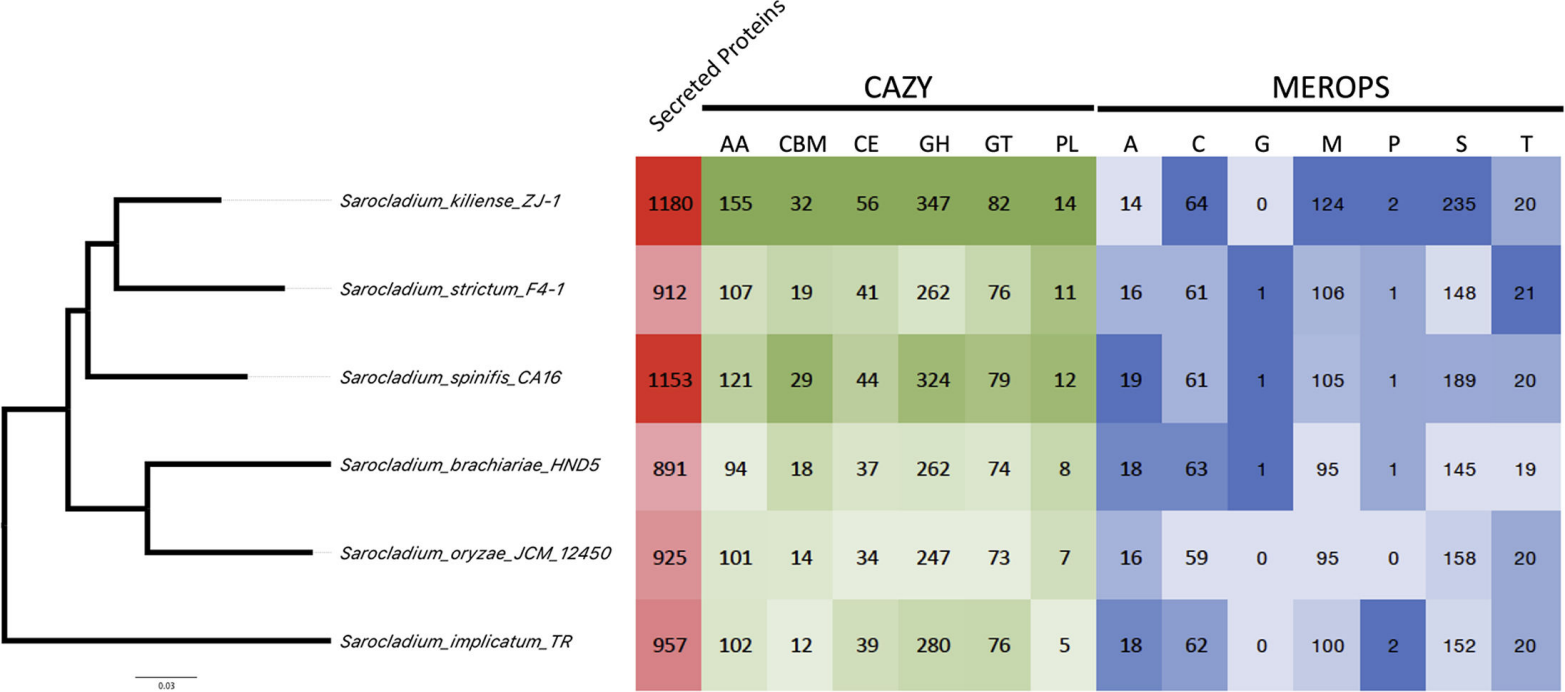
Phylogenomic relationships based on whole-genome sequence data. Phylogenomic relationships among *Sarocladium* species were inferred using 758 conserved protein markers extracted via the PHYling pipeline. This approach, which provides greater phylogenetic resolution than MLST analysis, confirmed that *S. spinificis* CA16 shares a common ancestor with *S. kiliense* ZJ-1 and *S. strictum* F4-1. Heat maps represent functional annotations for CAZy/dbCAN v.11.0 (carbohydrate-active enzymes, which are AA, auxiliary activity; CBM, carbohydrate binding module; CE, carbohydrate esterase; GH, glycoside hydrolase; GT, glycosyl transferase; PL, polysaccharide lyases), MEROPS v.12.0 (proteases, which are A, aspartate peptidase; C, cytosine peptidase; G, glutamic peptidase; M, metallopeptidase; P, mixed peptidase; S, serine peptidase; T, threonine peptidase), and secreted proteins. This highlights potential metabolic differences among *Sarocladium* species, with darker colors indicating more of that category relative to the other species analyzed.

We also used a phylogenomic approach based on 758 protein markers to infer the phylogenetic relationships between *Sarocladium* lineages. This analysis included fewer isolates but had more phylogenetic power than our MLST analysis. *S. spinificis* CA16 shares a common ancestor with *S. kiliense* ZJ-1 and *S. strictum* F4-1 (Fig. 4). This disagrees with the MLST analysis in Fig. 3 but could be due to a smaller number of taxa with single representatives for each species. There were six whole genomes of *Sarocladium* species of sufficient quality at the time of the analysis. Thus, future research will need to address the sources of discordance between the MLST and WGS phylogenetic analyses.

### *Sarocladium spinificis* inhibits the growth of *Coccidioides posadasii*

**Fig 5:**
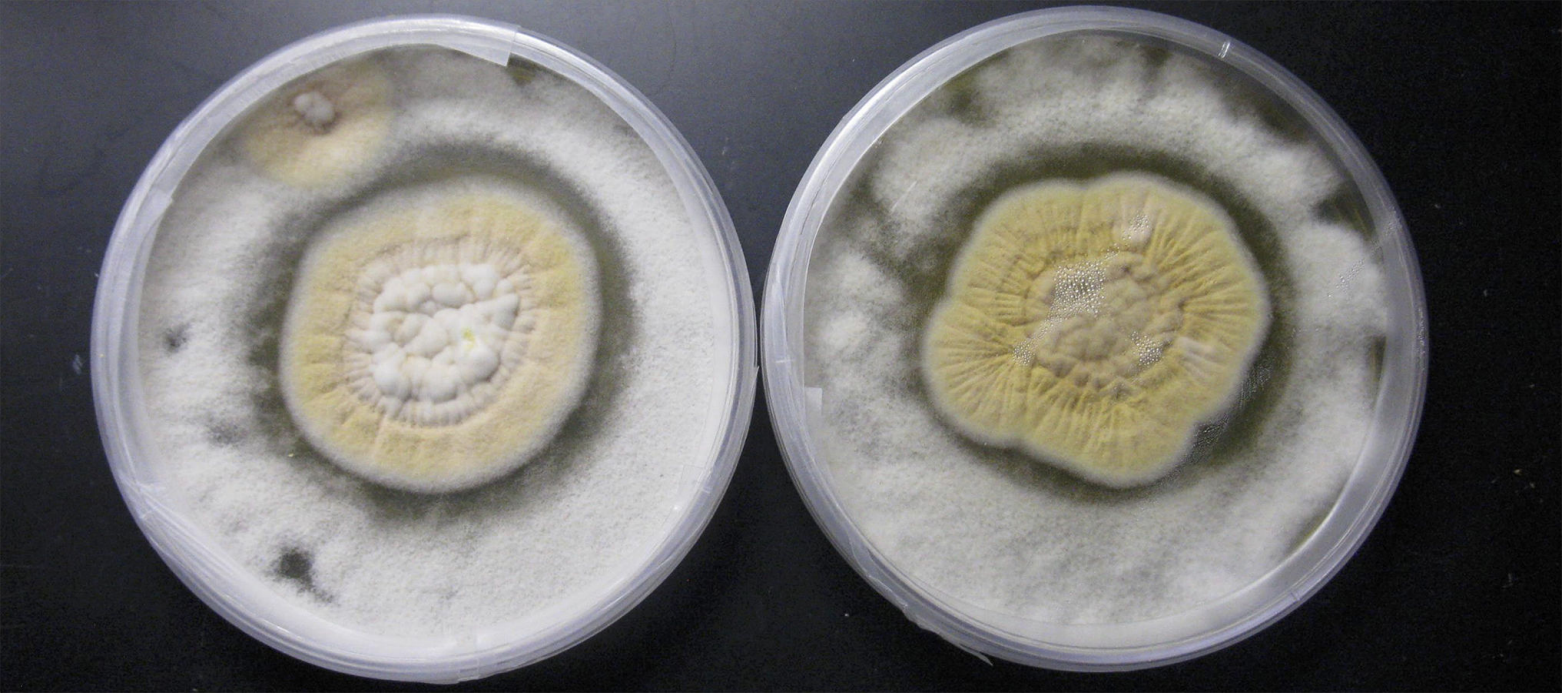
Antagonistic interactions between C*occidioides posadasii* and *Sarocladium spinificis*. After 10 days of growth, a distinct halo of inhibition was observed between the margin of *S. spinificis* CA16 or CA18 and *Coccidioides posadasii* NR-166, suggesting that *S. spinificis* is capable of inhibiting or restricting the growth of *C. posadasii*.

Both patients that were the source of strains CA16 and CA18 were diagnosed with coccidioidomycosis. Accuprobe testing confirmed *Coccidioides* spp. in the cultures initially, but morphological and qPCR analyses did not confirm the clinical diagnosis after storage. Interestingly, *S. spinificis* can grow at 37°C with macromorphological characteristics that resemble, but do not entirely match, those of *Coccidioides*. These factors make it challenging to determine whether the recovery of *S. spinificis* was a co-infection, a misdiagnosed case of coccidioidomycosis, or a thermotolerant contaminant. To begin to understand how those microorganisms interact, *C. posadasii* and *S. spinificis* were co-cultivated. After 10 days of growth, a distinct halo of inhibition was observed between the margin of both *S. spinificis* CA16 and CA18 with *C. posadasii* NR-166, suggesting that *S. spinificis* can inhibit, or at least restrict, the growth of *C. posadasii* (Fig. 5).

### Whole-genome analysis and annotation

The genomes of *S. spinificis* CA16 and CA18 were assembled into 414 and 368 contigs and yielded 33.61 and 33.73 Mb, respectively (Table 2). Despite their fragmentation, the genomes are largely complete according to BUSCO analysis (CA 16, 98%, and CA18, 98.2%; Table 2). The content of repetitive DNA in the *S. spinificis* genome was lower (CA16, 2.34%, and CA18, 2.47%) than that of other *Sarocladium* genomes (Table 2), such as *S. kiliense* (7.04%) and *S. strictum* (8.49%). In terms of the number of protein-coding genes, *S. kiliense* contains 12,635, while *S. spinificis* CA16 and CA18 strains have 11,023 and 10,935, respectively. The other *Sarocladium* species have a lower number of predicted genes (Table 2). Similarly, *S. kiliense* has 10,351 orthologous clusters and 1,648 singletons; *S. spinificis* has 9,952 orthologous clusters and 832 singletons; and the remaining *Sarocladium* species have fewer orthologous clusters and singletons.

**TABLE 2:** Summary of whole-genome assemblies and annotation of the *Sarocladium* species^*a*^

	*Sarocladium spinificis* CA16	*Sarocladium spinificis* CA18	*Sarocladium kiliense* ZJ-1	*Sarocladium strictum* F4-1	*Sarocladium brachiariae* HND5	*Sarocladium oryzae* JCM 12450	*Sarocladium implicatum* TR
**Assembly**							
# of contigs	414	368	51	12	19	47	17
Genome size (bp)	33,617,434	33,732,355	37,625,230	32,378,563	31,862,451	32,403,370	30,180,565
Largest contig (bp)	616,115	639,124	7,198,218	7,928,627	5,251,163	5,810,324	4,603,546
Repetitive DNA (%)	2.34	2.47	7.04	8.49	8.95	9.91	8.16
GC (%)	52.01	51.89	52.89	52.76	52.04	53.13	53.97
N50	169,435	176,882	2,236,409	4,214,981	3,269,626	3,262,506	3,477,858
L50	65	61	6	3	4	4	4
**Annotation**							
Gene models	11,136	11,048	12,635	9,944	9,721	10,188	9,950
mRNA	11,023	10,935	12,534	9,792	9,587	10,073	9,853
tRNA	113	113	101	152	134	115	97
Intron	18,988	19,482	21,901	16,886	16,844	17,397	17,547
Exons	30,011	30,417	34,435	26,678	26,431	27,470	27,400
Ave. exon length (bp)	475.78	474.06	462.24	478.86	486.51	479.63	473.68
Ave. gene length (bp)	1,622.28	1,652.25	1,582.35	1,630.12	1,688.09	1,640.78	1,649.53
Ave. protein length (aa)	500.01	507.01	483.9	502.97	515.54	500.2	505.72
**Functional**							
go_terms	2,807	2,756	2,615	2,077	2,017	2,806	2,791
InterProScan	3,338	3,279	3,122	2,431	2,353	3,272	3,269
eggNOG	10,573	10,484	11,889	9,417	9,253	9,574	9,458
Pfam	8,166	8,150	9,020	7,311	7,142	7,347	7,393
CAZymes	577	580	645	491	473	460	493
MEROPS	403	406	464	358	349	354	359
Secretion	1,153	1,161	1,180	912	891	925	957
Busco	3,754	3,760	3,766	3,728	3,709	3,738	3,721
Busco % completeness	98	98.2	98.3	96.8	96.9	97.4	97.3

^*a*^ CAZyme, carbohydrate-activated enzyme.

**Fig 6:**
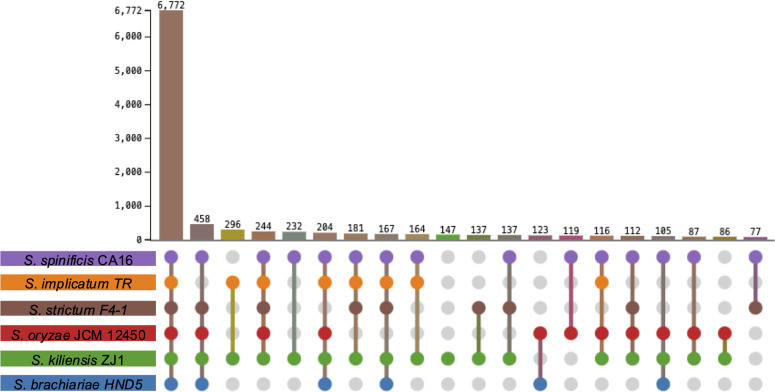
Analysis of shared gene content for six *Sarocladium* species. Orthologous cluster analysis was performed using OrthoVenn3, visualizing shared and unique protein clusters among *Sarocladium* genomes. Lines connecting circles indicate shared orthologous clusters for those species, and colors reflect each species.

In total, we found 6,772 shared orthologous clusters across the six *Sarocladium* species (Fig. 6). We also found 29 unique orthologous clusters for *S. spinificis* and according to GO analysis that are related to lipid and carbohydrate metabolism, cellular aromatic compound metabolic process (i.e., terpene), and transport (biological process) (Table S2). The only molecular function categories that were overrepresented were monooxygenase and oxidoreductase activities. Genes involved in the response to acidic pH (biological process GO:0010447) were significantly enriched in S. *spinificis* compared to the other species (*P* value = 0.0014).

We found similarities in protein family evolution between *S. kiliense* and *S. spinificis*, which differentiated them from other *Saracocladium* genomes (Fig. 6). We found an increase in eggNOG terms, Pfam, carbohydrate-activated enzymes (CAZymes), MEROPS (peptidases), and secreted proteins in *S. kiliense* and *S. spinificis* compared to *S. strictum*, *S. brachiariae*, and *S. oryzae*, which might reflect their different ecological roles and physiology (Fig. 4 and 6, Table 2). We found 232 shared orthologous clusters that have undergone gene expansion in both *S. kiliense* and *S. spinificis*, but not in other species. The genomic similarities between these two species are noteworthy because S. *kiliense* and *S. spinificis* have been implicated in opportunistic infections in humans, and both can grow at 37°C, a characteristic common to most fungal human pathogens (Fig. 2).

Our analysis identified expanded CAZy gene families in *S. kiliense* and *S. spinificis*. These included eight glycoside hydrolase (GH) families (GH18, GH43, GH16, GH5, GH78, GH35, GH109, and CBM91), a monooxygenase family (AA19), a glucose-methanol-choline oxidoreductase family (AA3), and four peptidase families (SX09, S10, S33, and M14B), all of which were enriched in their genomes (Fig. 4). MEROPS M38 protein family, a peptidase family of isoaspartyl dipeptidases, is highly expanded in *S. kiliense* (Fig. 4), and members of this family are known to play a role in virulence and pathogenesis in different fungal pathogens (54, 55).

We found a similar trend in transcription factors, indicated by gene family expansions of genes with TF domains in *S. kiliense* and *S. spinificis*. IPR007219, a fungal-specific TF domain, was enriched in the *S. kiliense* and *S. spinificis* genomes compared to the other *Sarocladium* species. Two TF domains showed expansions in plant pathogens. IPR000679, a GATA zinc finger domain, was enriched in *S. implicatum*, and IPR004827, a basic region leucine zipper 2 domain (bZIP TF 1), was enriched in *S. oryzae* (Fig. S2).

**Fig 7:**
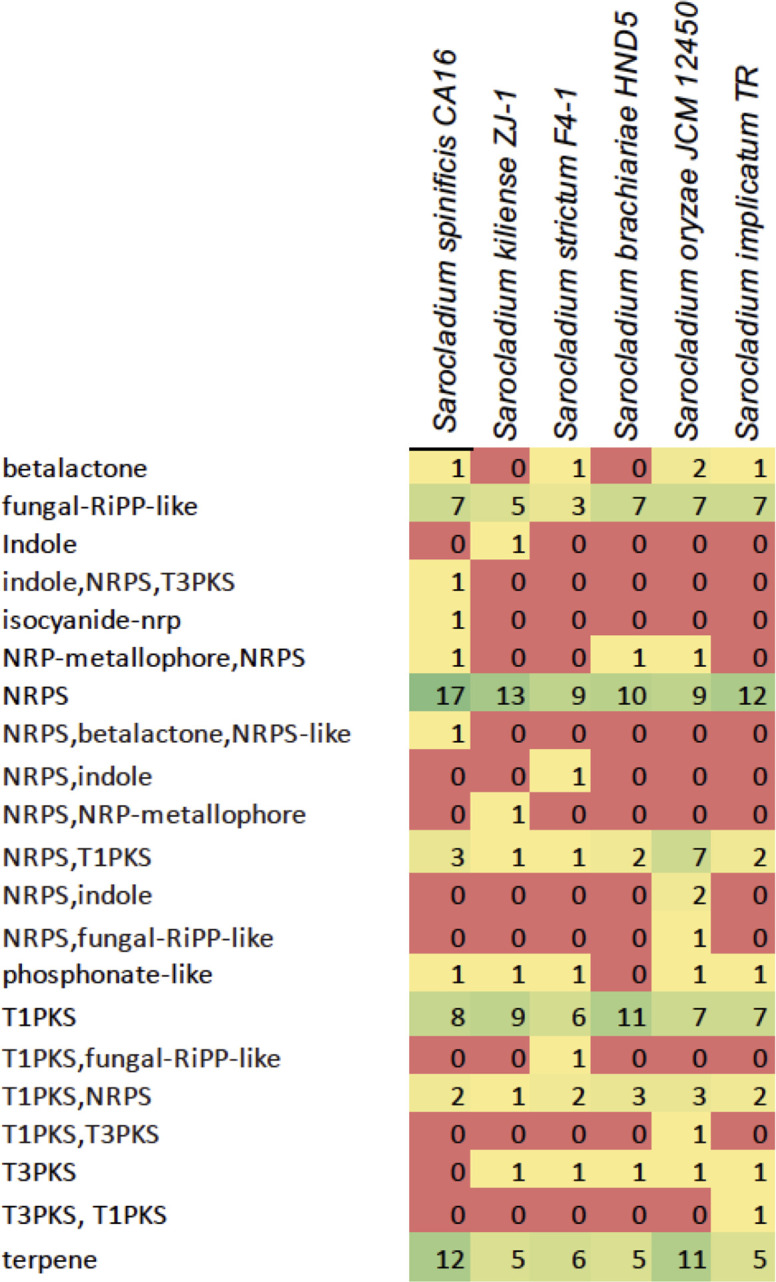
Expansion of secondary metabolite clusters among six species of *Sarocladium*. Secondary metabolite biosynthetic potential was assessed using antiSMASH, revealing key differences in the presence and composition of biosynthetic gene clusters involved in metabolite production across *Sarocladium* species. Green and dark green indicate expanded gene clusters.

We annotated 21 secondary metabolite gene groups in the six *Sarocladium* genomes (Fig. 7) and found notable differences. The indole-NRPS-T3PKS, NRPS-betalactone-NRPS-like, and isocyanide-nrp clusters are uniquely present in *S. spinificis* genomes, while the T3PKS cluster is absent compared to other *Sarocladium* species. We also observed a higher number of NPRS and terpene clusters in the *S. spinificis* genome in agreement with GO enrichment analysis (Fig. 7).

## DISCUSSION

In this study, we report that *Sarocladium spinificis* can be associated with human infections. We genomically characterized two strains, CA16 and CA18, both recovered from patients initially diagnosed with coccidioidomycosis based on clinical presentation as well as morphological and molecular diagnostics. After single spore isolation from glycerol stocks, molecular assays ruled out *Coccidioides* species and confirmed that these strains belonged to the genus *Sarocladium*. Identification was further refined through ITS locus amplification and sequencing, supported by phylogenetic analysis of multiple genetic loci, including whole-genome analyses. These analyses revealed a close relationship between CA16, CA18, and isolates of *S. spinificis* derived from plant sources. Previous reports have also identified *Parengyodontium* species, another plant pathogen, in patients diagnosed with coccidioidomycosis, indicating that there may be other potential fungal pathogens being masked by a more common fungal disease (56). The definite classification of these fungi as contaminants, opportunistic pathogens, or primary fungal pathogens remains an open question for further investigation. Regardless of the true ecological relationship between *Coccidioides* and *Sarocladium*, our results indicate the two species may co-occur in patients. We also report that these two *S*. *spinificis* restrict the growth of *Coccidioides*. Our findings contribute to the understanding of *S. spinificis* in two key aspects: as a human commensal capable of becoming an opportunistic pathogen and as a fungus that can suppress the growth of a major human pathogen.

*Sarocladium* species have been recently recognized as human pathogens, and little is known regarding their prevalence, or the molecular underpinnings behind virulence in mammals. The pathogen *S. kiliense* caused an outbreak of bloodstream infections in Chile and Colombia associated with contaminated antinausea medication administered to immunosuppressed patients (16). All isolates were closely related, which confirmed a single infectious source (16). It is worth noting that species of *Acremonium*, the former genus of *S. kiliense*, have also been implicated in human disease (reviewed in reference 8). Although our *S. spinificis* isolates were recovered from human patients, we cannot conclusively determine whether this species causes disease in humans or other mammals. Nonetheless, *S. spinificis*’ growth at 37°C is notable, suggesting that these fungi could survive in human host body temperature. The ability of these fungi to grow at this temperature indicates the possibility that they might emerge as pathogens adapting to higher temperatures under climate change and may be more likely to cause subcutaneous mycotic infections (57). We also report antifungal resistance in our two isolates of *S. spinificis* (Table 1), which is consistent with observations in *S. kiliense* (58). Experimental infections in mice or human cell lines are necessary to establish whether *S. spinificis*, like its close relative *S. kiliense*, has the potential to become a human health threat.

Our findings also provide new insights into the evolution of *Sarocladium*. Phylogenetic analysis of multiple loci confirmed that CA16 and CA18 are closely related to *S. spinificis* but not to *S. kiliense* (Fig. 3). This raises the question of whether other *Sarocladium* species can also cause disease or persist as human commensals, or if virulence mechanisms evolved independently in these species. Comparative genomics revealed that *S. spinificis* and *S. kiliense* have several shared secreted proteins, CAZymes, and peptidases, which may contribute to their ability to adapt to diverse ecological niches and their potential pathogenicity (Fig. 4). Additionally, the presence and diversity of monooxygenases and oxidoreductases suggest metabolic versatility. These traits appear to have evolved in parallel, indicating similar selective pressures (Table S1). These findings have broader implications. Determining the evolutionary mechanisms that have driven the evolution of virulence in the *Sarocladium* clade will require the resolution of the phylogenetic relationships between the species in the group using genome-wide markers.

The results of the co-cultivation experiments with *S. spinificis* and *C. posadasii* were particularly striking, as CA16 and CA18 originated from suspected coccidioidomycosis cases. Both strains inhibited *C. posadasii,* suggesting potential competitive interactions or the production of antifungal compounds, although this was at room temperature with the mycelial phase. *Coccidioides* has a distinctive morphology in the host, and the *in vitro* conditions that cause this switch are not amenable to competition assessments. Similar antagonistic interactions have been observed in other members of the order Hypocreales, such as *Trichoderma* (59, 60), *Acremonium* (61, 62), and *Beauveria* (63, 64), which produce antifungal metabolites and enzymes. *Sarocladium brachiariae* produces volatile compounds able to inhibit the growth of *Fusarium oxysporum* f. sp. *cubense* (65).

These findings underscore the ecological and clinical relevance of *Sarocladium spinificis* in affecting microbial communities and influencing disease dynamics. Through genomic characterization of strains CA16 and CA18, our study reveals novel insights into their genetic architecture, microbial interactions, and evolutionary relationships. By broadening the understanding of *Sarocladium* diversity and pathogenic potential, this work establishes a foundation for future investigations into its environmental roles, clinical prevalence, and promising directions for biotechnology and drug discovery.

## Data Availability

Data have been deposited in NCBI under BioProject identifier PRJNA1092660. CA16 read data are available as SRX31048444. CA18 read data are available as SRX31048445.

## SUPPLEMENTAL MATERIAL

Fig. S1 spectrum.00689-25-s0001.pdf Filamentous growth of *Sarocladium spinificis* CA16 and CA18 strains.

Fig. S2 spectrum.00689-25-s0002.pdf Expansion of transcription factor gene families in *Sarocladium* species.

Table S1 spectrum.00689-25-s0003.xlsx GenBank accession numbers for the five loci analyzed in the *Sarocladium multilocus* data set.

Table S2 spectrum.00689-25-s0004.xlsx Specific clusters enriched in *S. spinificis* compared to other *Sarocladium* genomes.

ASM does not own the copyrights to Supplemental Material that may be linked to, or accessed through, an article. The authors have granted ASM a non-exclusive, world-wide license to publish the Supplemental Material files. Please contact the corresponding author directly for reuse.
