# Supplementary material for: Morphological and genetic characterization of novel Sarocladium spinificis strains in association with Coccidioides posadasii
Source: Microbiol Spectr. 2025 Dec 29;14(2):e00689-25. doi: 10.1128/spectrum.00689-25 (PMC12889048; doi:10.1128/spectrum.00689-25)
Supplement: Fig. S1 — Filamentous growth of Sarocladium spinificis CA16 and CA18 strains. [file spectrum.00689-25-s0001.pdf]

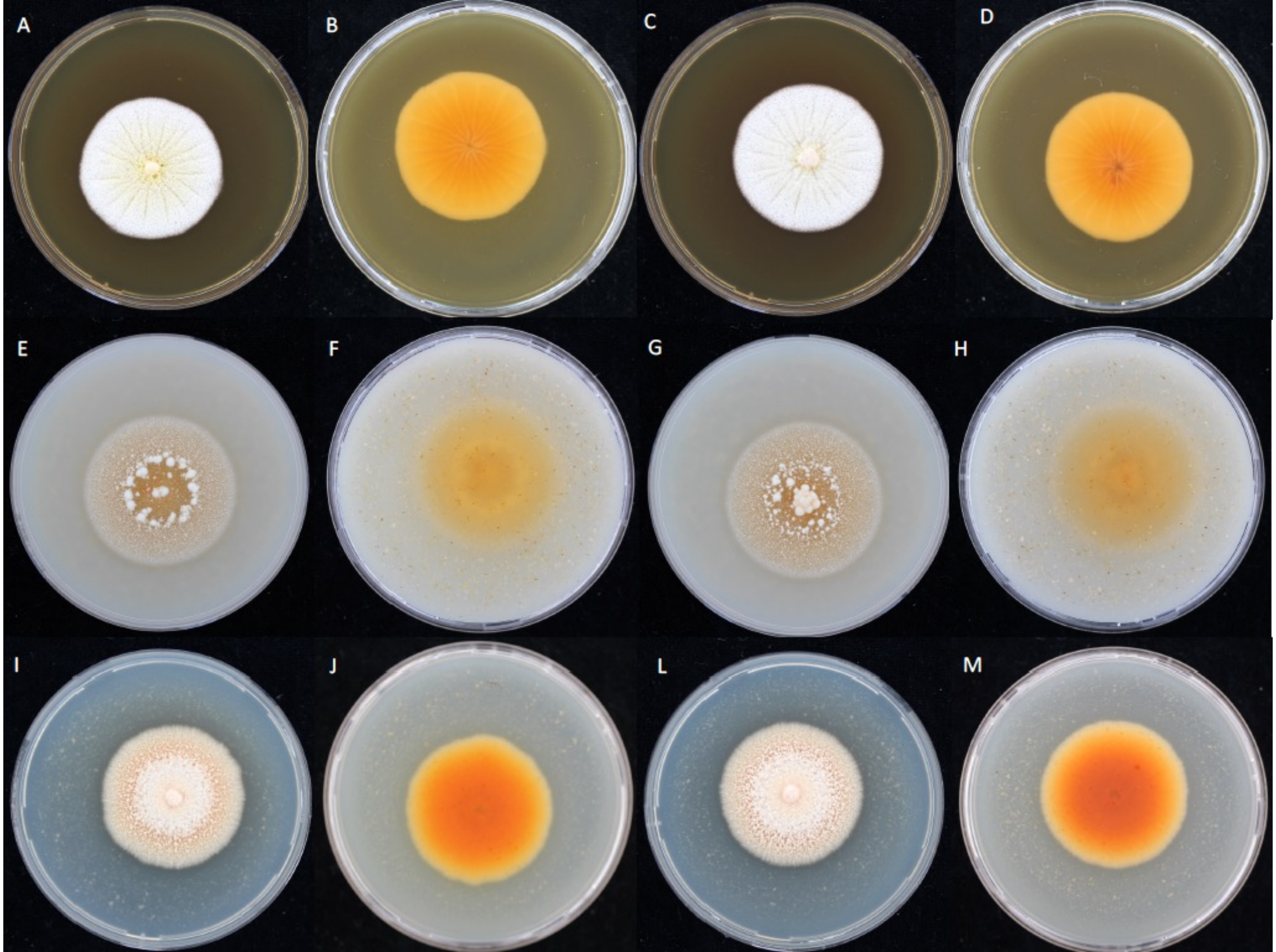
A
B
C
D
E
F
G
H
I
J
L
M

Figure S1. Filamentous growth of *Sarocladium spinificis* CA16 and CA18 strains. Fungal cultures were grown at 24°C on Potato Dextrose Agar (CA16, A-B and CA18, C-D), Oatmeal Agar (CA16, E-F and CA18, G-H) and Malt Extract Agar (CA16, I-J and CA18, L-M showing similar macromorphology with little phenotypic variability. Colonies circular, regular, flat, whitish or creamy buff, sometimes radially or slightly sulcate. In all media, yellow to orange pigment was produced.
