# Supplementary material for: Morphological and genetic characterization of novel Sarocladium spinificis strains in association with Coccidioides posadasii
Source: Microbiol Spectr. 2025 Dec 29;14(2):e00689-25. doi: 10.1128/spectrum.00689-25 (PMC12889048; doi:10.1128/spectrum.00689-25)
Supplement: Fig. S2 — Expansion of transcription factor gene families in Sarocladium species. [file spectrum.00689-25-s0002.pdf]

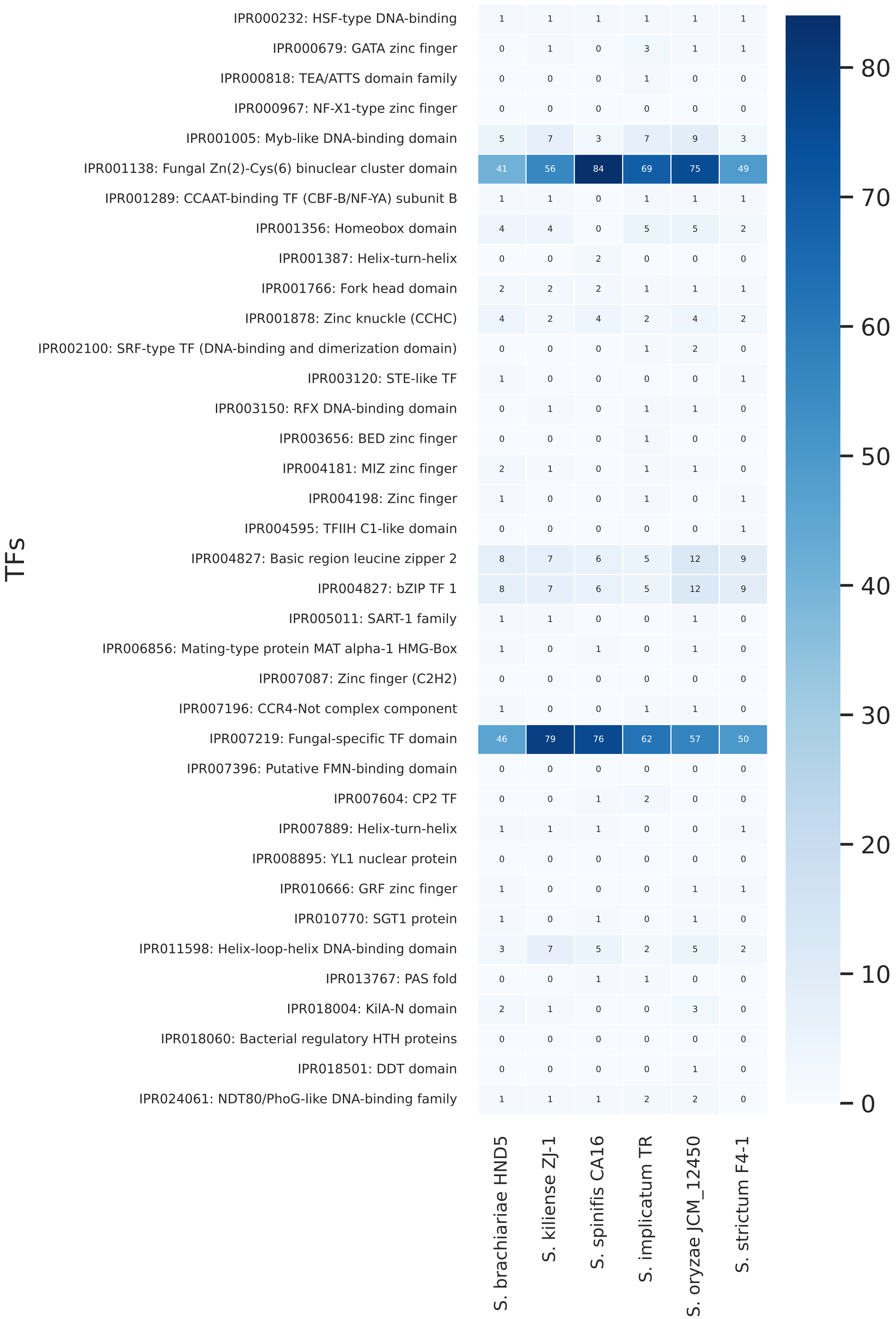
TFs
IPR000232: HSF-type DNA-binding 1 1 1 1 1 1
IPR000679: GATA zinc finger 0 1 0 3 1 1
IPR000818: TEA/ATTS domain family 0 0 0 1 0 0
IPR000967: NF-X1-type zinc finger 0 0 0 0 0 0
IPR001005: Myb-like DNA-binding domain 5 7 3 7 9 3
IPR001138: Fungal Zn(2)-Cys(6) binuclear cluster domain 41 56 84 69 75 49
IPR001289: CCAAT-binding TF (CBF-B/NF-YA) subunit B 1 1 0 1 1 1
IPR001356: Homeobox domain 4 4 0 5 5 2
IPR001387: Helix-turn-helix 0 0 2 0 0 0
IPR001766: Fork head domain 2 2 2 1 1 1
IPR001878: Zinc knuckle (CCHC) 4 2 4 2 4 2
IPR002100: SRF-type TF (DNA-binding and dimerization domain) 0 0 0 1 2 0
IPR003120: STE-like TF 1 0 0 0 0 1
IPR003150: RFX DNA-binding domain 0 1 0 1 1 0
IPR003656: BED zinc finger 0 0 0 1 0 0
IPR004181: MIZ zinc finger 2 1 0 1 1 0
IPR004198: Zinc finger 1 0 0 1 0 1
IPR004595: TFIIH C1-like domain 0 0 0 0 0 1
IPR004827: Basic region leucine zipper 2 8 7 6 5 12 9
IPR004827: bZIP TF 1 8 7 6 5 12 9
IPR005011: SART-1 family 1 1 0 0 1 0
IPR006856: Mating-type protein MAT alpha-1 HMG-Box 1 0 1 0 1 0
IPR007087: Zinc finger (C2H2) 0 0 0 0 0 0
IPR007196: CCR4-Not complex component 1 0 0 1 1 0
IPR007219: Fungal-specific TF domain 46 79 76 62 57 50
IPR007396: Putative FMN-binding domain 0 0 0 0 0 0
IPR007604: CP2 TF 0 0 1 2 0 0
IPR007889: Helix-turn-helix 1 1 1 0 0 1
IPR008895: YL1 nuclear protein 0 0 0 0 0 0
IPR010666: GRF zinc finger 1 0 0 0 1 1
IPR010770: SGT1 protein 1 0 1 0 1 0
IPR011598: Helix-loop-helix DNA-binding domain 3 7 5 2 5 2
IPR013767: PAS fold 0 0 1 1 0 0
IPR018004: KilA-N domain 2 1 0 0 3 0
IPR018060: Bacterial regulatory HTH proteins 0 0 0 0 0 0
IPR018501: DDT domain 0 0 0 0 1 0
IPR024061: NDT80/PhoG-like DNA-binding family 1 1 1 2 2 0
S. brachiariae HND5
S. kiliense ZJ-1
S. spinifis CA16
S. implicatum TR
S. oryzae JCM_12450
S. strictum F4-1
80
70
60
50
40
30
20
10
0

Figure S2. Expansion of Transcription Factor Gene Families in *Sarocladium* Species. We identified transcription factor (TF) gene family expansions in *Sarocladium kiliense* and *Sarocladium spinificis* using genome data from CA16, CA18, and previously sequenced *Sarocladium* genomes. To analyze genomic trends in annotated TF categories, we employed the Funannotate v1.8 compare function across six *Sarocladium* species. Our analysis revealed that IPR007219, a fungal-specific TF domain, was significantly enriched in *S. kiliense* and *S. spinificis* compared to other *Sarocladium* species. Additionally, we observed expansions of transcription factors associated with plant pathogens. Specifically, IPR000679 (GATA zinc finger domain) was enriched in *S. implicatum*, while IPR004827 (basic region leucine zipper 2 domain, bZIP TF 1) was significantly enriched in *S. oryzae.*
